# Perceptual Anchoring in Preschool Children: Not Adultlike, but There

**DOI:** 10.1371/journal.pone.0019769

**Published:** 2011-05-16

**Authors:** Karen Banai, Rachel Yifat

**Affiliations:** Department of Communication Sciences and Disorders, University of Haifa, Haifa, Israel; Ecole Polytechnique Federale de Lausanne, Switzerland

## Abstract

**Background:**

Recent studies suggest that human auditory perception follows a prolonged developmental trajectory, sometimes continuing well into adolescence. Whereas both sensory and cognitive accounts have been proposed, the development of the ability to base current perceptual decisions on prior information, an ability that strongly benefits adult perception, has not been directly explored. Here we ask whether the auditory frequency discrimination of preschool children also improves when given the opportunity to use previously presented standard stimuli as perceptual anchors, and whether the magnitude of this anchoring effect undergoes developmental changes.

**Methodology/Principal Findings:**

Frequency discrimination was tested using two adaptive same/different protocols. In one protocol (*with-reference*), a repeated 1-kHz standard tone was presented repeatedly across trials. In the other (*no-reference*), no such repetitions occurred. Verbal memory and early reading skills were also evaluated to determine if the pattern of correlations between frequency discrimination, memory and literacy is similar to that previously reported in older children and adults. Preschool children were significantly more sensitive in the with-reference than in the no-reference condition, but the magnitude of this anchoring effect was smaller than that observed in adults. The pattern of correlations among discrimination thresholds, memory and literacy replicated previous reports in older children.

**Conclusions/Significance:**

The processes allowing the use of context to form perceptual anchors are already functional among preschool children, albeit to a lesser extent than in adults. Nevertheless, immature anchoring cannot fully account for the poorer frequency discrimination abilities of young children. That anchoring is present among the majority of typically developing preschool children suggests that the anchoring deficits observed among individuals with dyslexia represent a true deficit rather than a developmental delay.

## Introduction

An intriguing characteristic of human perception is that perceptual skill continues to improve well into adolescence despite the relatively early maturation of the sensory neural pathways themselves [1], [2]. While this dissociation has often been interpreted to suggest that non-sensory factors (e.g., attention) are responsible for the prolonged development of perceptual skill, the processes contributing to this prolonged development remain poorly understood. One process that has been shown to strongly influence adult perception, is anchoring (or more generally, predictive coding) – the implicit ability to use the contextual information embedded in past stimuli to guide subsequent performance [3], [4]. The tasks used to measure perceptual skills in children are often those that produce large anchoring effects in adults. Therefore, a plausible hypothesis is that anchoring is one of the processes that contribute to the prolonged development of perceptual skill. Whether anchoring influences performance in young children and if so whether it is mature remains unknown. The major goals of the current study were therefore to determine whether anchoring influences auditory frequency discrimination in preschool children and to compare the magnitude of the effect to that observed in adults, using the same, child friendly, assessment procedure. A secondary goal, deriving from the suggestion that anchoring is related to memory, was to test whether the same relationships between anchoring and memory are observed in young children as in adolescents and adults [5], [6], [7].

Studying the effects of anchoring on the discrimination skills of young children and the relationships between anchoring and memory, is of interest not only because it can shed light on the factors contributing to perceptual development beyond infancy, but also because it has been hypothesized (see [6] for a recent review) that impaired anchoring may contribute to the development of reading difficulties in school age children. If this is the case, anchoring should be observed among typically developing children prior to school entry and the onset of formal reading instruction. Furthermore, it is expected to be impaired among children who are at risk of developing reading difficulties (although this question is beyond the scope of the current study).

In adults and adolescents, the anchoring effect was studied rather extensively for auditory frequency discrimination [3], [5], [8], [9]. In a typical frequency discrimination experiment, listeners are presented, on each trial, with two consecutive tones and are asked to determine which of the two is higher in pitch. The initial frequency difference between the two tones is large and is subsequently adjusted based on performance until a discrimination threshold is reached. This adaptive procedure can be implemented with different testing protocols. In a *repeated reference* protocol, a fixed reference tone (e.g., 1000 Hz) is presented on each and every trial. Thus, if the initial frequency difference is 200 Hz, the frequency of the other tone on the first trial is 1200 Hz and it subsequently decreases as long as the listener continues to respond correctly. In a *no reference* protocol, one of the stimuli on each trial is randomly selected from a pre-determined frequency interval (e.g., 800–1200 Hz). Therefore, if a 1100 Hz tone is selected on the first trial, and the starting frequency difference is 200 Hz, the other tone is 1300 Hz. A consistent finding is that discrimination thresholds are significantly lower (better) when tested with repeated reference protocols than when tested with no reference ones. In other words, when listeners can use the across trial repetitions as ‘anchors’, discrimination at the single trial level improves, sometimes by an order of magnitude. While similar effects were observed across sensory modalities and in non-human species [10], [11], [12], [13] it is not known whether child perception is similarly sensitive to context or whether poorer perception in children can be attributed, at least in part, to immature anchoring mechanisms. There is evidence that the infant brain is sensitive to the context of recently presented stimuli when measured in passive listening paradigms [14], [15], [16]. For example, physiological mismatch responses to occasional pitch changes within sound sequences were observed among 4 months old infants [14], suggesting that their brains are sensitive to the structure of the sequence. Whether young children can use this physiological sensitivity to guide their conscious perception is still unclear.

Previous developmental investigations of auditory frequency discrimination [17], [18], [19], [20], [21] suggest that the age in which discrimination reaches adult level greatly depends on the assessment protocol, with some procedures not yielding adult like performance even by 11 years of age [20]. Determining whether anchoring plays a role in the poor discrimination capacities of young children based on those previous studies is difficult, because only a single assessment protocol was typically used, or several protocols were used, but the study was not designed to directly test the effects of anchoring [19]. Nonetheless, the finding that discrimination thresholds of 6–7 years old children improved when the number of fixed reference stimuli per trial increased [19] suggests that children of this age are probably able to benefit from anchoring. However whether the degree of benefit is similar to that observed in adults and whether it relies on repetition within or across trials has not been determined. The current study was therefore designed to directly test the hypothesis that preschool children manifest *across trial* anchoring in a frequency discrimination task. We used two versions of a two-interval two-alternative same/different task, one in which a single reference tone was repeated in a fixed temporal position across trials (*‘with reference’*) and one with no across trial repetitions at all (*‘no-reference’*), and compared the performance of preschool children to that of adults. While frequency discrimination thresholds in children as young as four years of age were reported before [17], [21], this is the first study, to our knowledge to test the effects of non-sensory factors that are related to the dynamics of the assessment protocol in this age group.

## Methods

### Participants

Children: Ninety four typically developing children participated in the study. Seventeen children did not complete at least one of the frequency discrimination tasks (see below) due to time constraints (n = 7), excessive background noise during testing (n = 6), or because the child asked to discontinue the test (n = 4) and their data were excluded from the current report. Therefore we report data from 77 children (34 girls), aged 50–78 months (average ± s.d.: 66±5). By parental reports all children were native monolingual Hebrew speakers and were never diagnosed with any neurological, developmental, hearing or cognitive disorder. None of the children had first degree relatives diagnosed with a reading, language or learning disability. All children came from communities of average or above average socioeconomic status in northern and central Israel and attended municipal preschools/kindergartens in their communities. By teacher evaluations all children were normally achieving in terms of the kindergarten curriculum and no concerns were expressed regarding their academic status.

Adults: The frequency discrimination data of the children were compared to that of 20 young adults (10 females, mean age: 25±1.6 years) who participated in a previous study on frequency discrimination in adults and tested in environmental conditions similar to those in which the children were tested, that is outside the lab in university classes during breaks, the dorms etc. [22].

A written informed consent was obtained from all adult participants and from the parents of all participating children prior to study onset. All aspects of this study were approved by the ethics committee of the Faculty of Social Welfare and Health Sciences at the University of Haifa as well as by the chief scientist of the Israeli Ministry of Education.

### Procedure

Children were tested individually in quiet areas of their schools by female research assistants with training in Communication Sciences and Disorders or in Education. Each session lasted approximately 40 minutes (including the introduction, instructions and break periods) and comprised of a battery of frequency discrimination, verbal memory and early literacy tasks. The order of the different tasks was counterbalanced across children.

### Tasks

#### Frequency Discrimination

Frequency discrimination was measured in two conditions (*with-reference* and *no-reference*) using a two-interval two-alternatives forced choice same/different task. On half the trials, the two tones were identical (same trials). On the other half (different trials), the second tone was higher than the first. On each trial listeners were asked to determine whether the two tones were the same or different. The order of same and different trials was randomly determined. The frequency difference (ΔF) between the two tones on the first different trial was 500 Hz and it was adapted based on listeners' performance using a 3 down/1 up staircase procedure converging on a performance level of 79% [23]. Adaptation of the frequency difference was based on performance on different trials only. For the first 3 reversals, the frequency difference was halved or doubled following correct/incorrect responses. Subsequently the difference was divided or multiplied by a factor of 1.41. The stimuli were 200 ms pure tones with inter-stimulus intervals of 500 ms. 100 trials were administered on each condition.

In the *with-reference* condition the two stimuli on same trials and the first stimulus on different trials were always 1000 Hz tones while the second tone on different trials was higher (1000+ΔF Hz), with ΔF determined by the adaptive procedure. In the *no-reference* condition the first tone on each trial was randomly sampled from the 800–1200 Hz frequency range. On same trials the second tone was identical, on different trials the second tone was always of a higher frequency with the frequency difference (ΔF) being determined by the adaptive procedure. The two conditions were otherwise identical and the order of administration was counterbalanced across participants. The discrimination tasks were administered using a child friendly interface coded in Matlab which provided pleasant visual feedback in the form of a smiley cartoon following each correct response and a sad smiley cartoon following each incorrect one. After each trial the child indicated her response to the experimenter by pointing at the computer screen and the experimenter entered the response using a mouse. This mode of response was selected because we were concerned that the younger children may not be as proficient using a mouse, leading to errors that are not related to their discrimination ability. It also allowed the experimenter to determine that the child was attentive to the task. Adults selected their responses directly using a mouse.

Prior to the first condition, the experiment was presented to the children in stages. First, to verify that children were familiar with concepts of ‘same’ and ‘different’, they were presented with pictures of object pairs (e.g., two apples, an apple and a dog) and were asked to determine if the two objects were the same or not. Subsequently the experimenter played tone pairs and the children had again to determine if they were the same or not. None of the children in the current sample had difficulties in this phase. This phase was skipped for the adult participants. Second, after being satisfied that a child had no difficulty determining that two tones were identical or different, or in the case of adults, an example block of 5 trials with 1000 Hz frequency difference on the different trials was administered. If the participant responded correctly on at least 4 trials administration of the first adaptive condition begun. Otherwise, another example block was administered. Again, all 77 children and all adults passed this phase.

To be included in the final data set we required participants to have (1) an overall performance level of 55% correct or higher, and (2) a higher proportion of hits (correctly determining that different tones are different) than false alarms (incorrectly deciding that two tones on a same trial are different) which would be roughly equivalent to d prime ≥1. Five children failed to fulfil this criterion for at least one of the conditions and their data were excluded from further analysis. The final data set thus includes data from 72 children and 20 adults.

Frequency discrimination thresholds (JNDs) were calculated as the geometric mean of ΔF values in the largest even number of reversal trials after excluding the first 3 or 4 reversals. A reversal trial is defined as a trial in which ΔF changed from decreasing to increasing or vice-versa. Because JNDs were not normally distributed, statistical analyses were conducted on the logs of the JND values. These values were approximately normally distributed (Kolmogorov-Smirnov tests, p>0.2).

#### Verbal Memory

*Verbal memory span* was assessed with a syllable Span task. Children were required to repeat lists of syllables read by the experimenter in a pace of 1 syllable/sec. Syllables were ordered as to not produce any meaningful Hebrew words (e.g., /na/,/shi/,/do/). The first list was two items long and list length increased up to a maximum of 9 items per list. Two lists were presented at each list length and testing was discontinued if a participant failed to correctly repeat two lists of the same length. Only items in which all the syllables were repeated in the order of presentation counted as correct items. Final score ranged from zero to sixteen.

*Verbal working memory* (*‘antonyms’*, designed by Ben Dror and Shany). In this task, lists of two, three or four common adjectives (e.g., black, long) were presented and the participant was required to repeat the opposite of each adjective in order of presentation (e.g., black, short → white, long). Two lists were presented at each list length. Scoring: each correctly given antonym receives a point. In addition, for each list 1 extra point is given if all items were given in the correct order. Final scores thus could range from zero to 24. Prior to the onset of the test, the experimenter read each adjective to the child and made sure they could produce its antonym. All children could produce all antonyms.

#### Early Literacy

Early reading skills were estimated with the tasks described below to verify that children were showing age appropriate progress on those skills (based on the norms provided by the test creators) and to allow us to compare the relationships between frequency discrimination, verbal memory and phonological awareness to those previously reported in adolescents [5], [24].

*Phonological awareness* was assessed using two subtests from the battery developed by Tubul, Lapidot and Wohl [25] requiring phoneme identification at word initial or word final positions. On each trial, the experimenter presented one word (e.g. dog) and asked the child whether that word begun (or ended) with a particular phoneme (e.g., does the word dog begin with the sound /d/, to which the child should respond ‘yes’, or does the word cat end with the sound /m/ to which the child should respond ‘no’). Prior to the beginning of formal assessment that task was explained and demonstrated by the experimenter. Three sample items in which the experimenter provided feedback and corrected the child in case of an error were presented before each section of the test. If a child failed all three items, testing was discontinued. The final score was the average number of correct items in the two sections of the test and could range from zero to ten.

*Letter identification*. Familiarity with letter names was assessed by showing children a sheet on which all (22) letters of the Hebrew alphabet are printed in bold type font [26]. The letters were arranged on the sheet randomly. Children were asked to name all the letters they can recognize. Only production of the full name of the letter counted as a correct answer and therefore scores could range from zero to twenty two.

## Results

**Figure 1:**
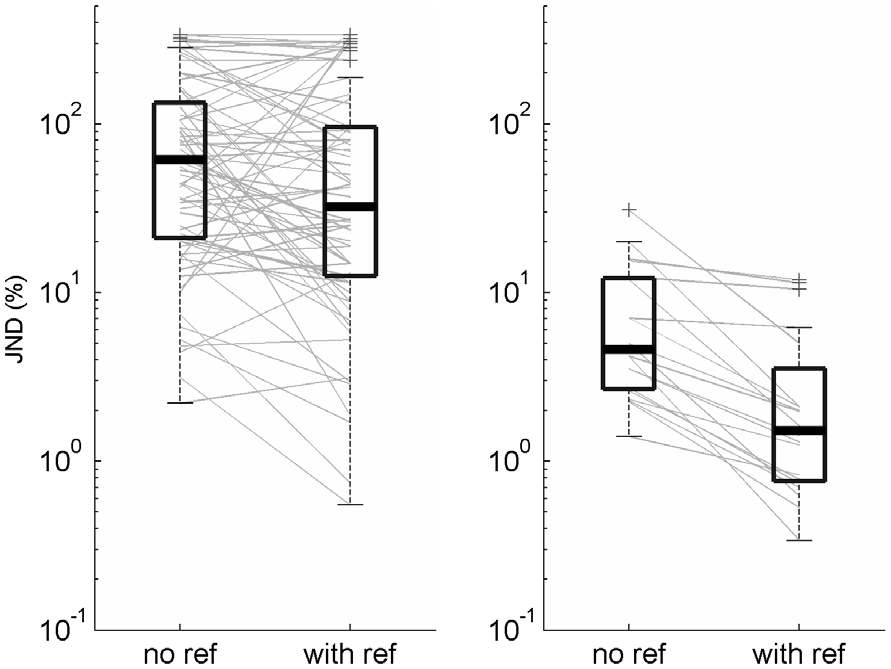
Frequency discrimination thresholds (JND) in children (left) and adults (right). Box edges mark the inter-quartile range, the black line within each box marks the group median, and whiskers are 1.5 times the inter-quartile range. Individual listeners' data is shown with thin gray lines connecting the two conditions.

**Figure 2:**
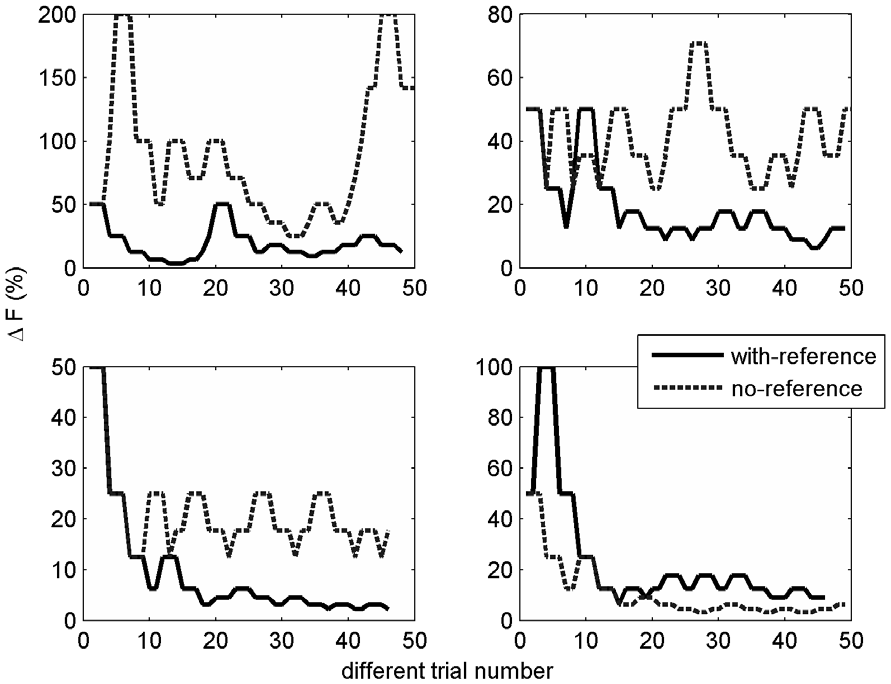
Examples of the frequency differences (in %) throughout the ‘different’ trials of the with reference (black line) and no reference (dashed line) conditions from 4 representative children. In three out of the four cases (top row and bottom left panels) frequency differences throughout the block are larger for the no reference condition. The final case (bottom right panel) represents the 30% of children in which no anchoring was observed.

Frequency discrimination thresholds (JNDs) of the child participants in the two discrimination conditions are shown in Figure 1 (left panel). Significant anchoring effects were evident among the preschool participants of the current study, consistent with previous findings in adolescents and adults. Discrimination thresholds in the *with-reference* condition were lower than those in the *no-reference* condition in 50 out of 72 (69%) children (compared with 50% as would have been expected if the difference between the conditions occurred at a random direction; Binomial test, p = 0.006, see Figure 1, grey lines denote individual data). Corresponding to the individual data, at the group level, discrimination thresholds were significantly lower in the reference containing than in the no-reference condition (a 2 conditions x 2 orders ANOVA with condition as a within-subject factor; F_condition_ = 8.51, p = 0.005, see Figure 1, box plots). There was no effect of the order in which the conditions were performed (F = 0.05, p = 0.82) nor did the order influence the anchoring effect (order x condition interaction: F = 0.94, p = 0.34). Examples of performance throughout the ‘different’ trials of each condition from 4 individual children are shown in Figure 2. Gender had no significant influence on either discrimination thresholds or on the anchoring effect as confirmed with a 2 conditions x 2 orders x 2 genders ANOVA with condition as a within-subject factor (F_condition_ = 5.96, p = 0.017, F_gender_ = 0.74, p = 0.48, F_order_ = 0.26, p = 0.62, all interaction effects insignificant with F<1.66, p>0.2). Likewise, age was not significantly correlated to discrimination thresholds in either condition (r = 0.16 and −0.09, for age and JNDs in the with reference and no-reference condition, respectively).

**Figure 3:**
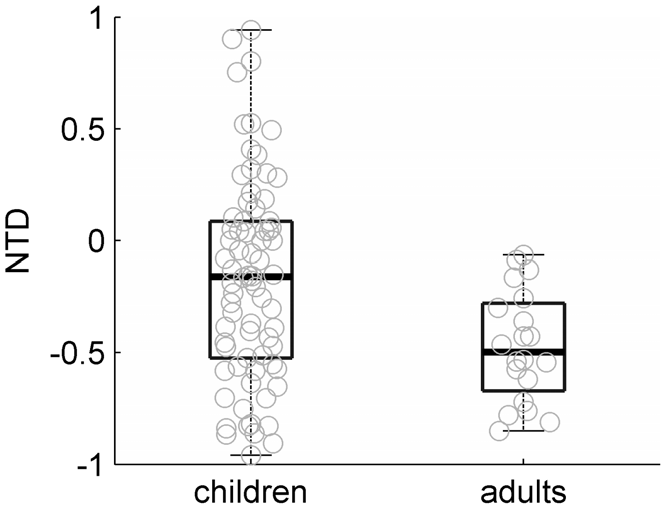
Normalized threshold differences (NTD) in children and adults. Negative values indicate an anchoring effect (see text for details). Boxes represent group data (see Figure 1 for details). Individual data is marked with gray circles.

Overall, the mean magnitude of the anchoring effect, or the normalized threshold difference (NTD) defined as the difference between JNDs in the *with-reference* and *no-reference* conditions divided by the sum of the JNDs in the two conditions was −0.18±0.46 (see Figure 3) and significantly smaller from zero (t = −3.26, p = 0.002). Taken together, these data suggest that the presence of a repeated reference across trials helps to improve performance even in young children.

The anchoring effect observed in the current experiment did not likely result from differences in the overall level of difficulty of the reference containing and the no-reference conditions. Thus, as shown in Table 1, there were no significant differences in the number of reversals obtained on each of the conditions or in the proportions of hits (‘different’ responses in ‘different’ trials) and false alarms (‘different’ responses in ‘same’ trials).

**Table 1:** Properties of psychophysical performance in each discrimination condition.

	With referenceMean (s.d)		No referenceMean (s.d)		F (p)^1^
	children	adults	children	adults	
Number of reversals	9.5 (2)	9.6 (2)	9.6 (2)	9.5 (2)	0.04 (0.84)
Proportion of hits	0.78 (0.07)	0.84^2^ (0.03)	0.77 (0.06)	0.81^2^ (0.03)	0.53 (0.49)
Proportion of false alarms	0.20 (0.14)	0.15 (0.09)	0.23 (0.15)	0.17 (0.10)	2.19 (0.14)

1 Main effect of condition in a repeated measures ANOVA conducted among children with condition (with-reference, no-reference) as a within listener and order as a between listener factor. The effect of order and the interaction terms also were insignificant.

2 Significant age effect in favor of adults (p<0.02) determined with 2-samples Kolmogorov-Smirnov tests.

### Comparison to adult data

Overall discrimination thresholds in both conditions were more than an order of magnitude higher (poorer) in children (mean±s.d: 87%±109 and 97%±100, in the *with- reference and no-reference conditions* respectively) than in adults (mean±s.d: 3%±4 and 8%±4, respectively), as shown in Figure 1 (F_age group_ = 74, p<0.001). Furthermore, while the main effect of condition was highly significant (F = 26, p<0.001), a significant interaction between age group and condition (F = 5.3, p = 0.024) suggests that the anchoring effect was susceptible to developmental changes. As was the case for the children's data alone, order had no effect on discrimination thresholds, nor did it interact with discrimination condition (F<0.8, p>0.33).

While both children and adults benefited from the opportunity to use stimulus regularities to guide perceptual discriminations, both the proportion of individuals benefiting from stimulus regularities and the magnitude of the effect appear to increase with age (see Figure 3). Thus, all adults in the current study had lower discrimination thresholds in the *with-reference* than in the *no-reference* condition (compared with 69% of the children). Furthermore, the mean NTD of adults (−0.47±0.25) was also significantly larger (in absolute terms) than that of the children (t(59.3) = 3.74, p<0.001; see Figure 3).

Finally it should be noted that it is unlikely that the age related differences in discrimination thresholds we report result from procedural factors associated with the discrimination task we used. Thus, neither the number of reversals, nor the proportion of false alarms differed significantly between children and adults for either condition (see Table 1). On the other hand, adults made significantly more hits than children on both the reference and the no reference discrimination conditions (see Table 1) consistent with their lower discrimination thresholds.

### Frequency discrimination and working memory in children

A significant (r = 0.48, p<0.01) correlation was observed between JNDs in the two conditions but the correlation is in no way perfect suggesting that different underlying processes may affect performance on each condition. This imperfect correlation suggests that the magnitude of the anchoring benefit induced by the availability of the repeated reference may be partially independent of the factors enabling good frequency discrimination in the no-reference condition.

Frequency discrimination in the no-reference condition was significantly associated with verbal working memory skill, but not with letter knowledge or phonological awareness (see Table 2). On the other hand, frequency discrimination in the reference containing condition as well as the degree of anchoring benefit were not correlated to any of the memory or reading related skills measured here, a pattern similar to that observed previously among typically developing adolescents [5]. Furthermore, verbal working memory continued to predict a significant amount of variance in the no-reference condition, even after the contribution of the with-reference condition was statistically accounted for in a regression model to which with-reference frequency discrimination was entered in the first step and verbal working memory was entered in the second step (see Table 3). While the proportion of independent variance in the no-reference frequency discrimination accounted for by verbal working memory is relatively small (6%), the strength of association between non-verbal frequency discrimination and verbal working memory is similar in magnitude to that observed among the different verbal tasks used in the current study (see Table 2) suggesting that verbal and non-verbal abilities may share specific but common processing bottlenecks that are related to working memory. Interestingly though, it would appear that perceptual anchoring and working memory make independent contributions to performance, at-least in this age group.

**Table 2:** Pearson correlations among study variables.

	PA	Letter Id	PM	WM	With-ref	No-ref
Age	0.07	0.04	0.01	0.17	0.16	−0.09
PA	–	**0.49** **	**0.53** **	**0.45** **	0.03	−0.12
Letter Id		–	0.25*	0.26*	0.09	−0.07
PM			–	0.21	−0.09	−0.13
WM				–	−0.21	**−0.34** **
With-ref					–	**0.48** **
No-ref						–

PA: phonological awareness, Letter Id: letter identification, PM: memory span, WM: working memory, with-ref: frequency discrimination with-reference, no-ref: frequency discrimination no-reference.

*p<0.5,

**p<0.01.

**Table 3:** Regression models for predicting no-reference frequency discrimination.

Predictors	R^2^	F (p)	β	t (p)	R^2^ change	F(p) change
**Step 1**	0.23	21.3 (<0.001)				
With-ref			0.49	4.61 (<0.001)		
**Step 2**	0.29	14.2 (<0.001)			0.06	5.63 (0.02)
With-ref			0.43	4.16 (<0.001)		
WM			−0.25	−2.37 (0.02)		

## Discussion

Irrespective of the discrimination condition used, frequency discrimination thresholds of preschool children in the current study were far from adult-like, as would have been expected based on previous studies. Nevertheless, the presence of across trial stimulus repetitions significantly improved discrimination capacity among children, albeit the degree of improvement was smaller than in adults. These findings suggest that while anchoring mechanisms are functional among typically developing preschool children and can be used to guide conscious perception, the benefit they provide continues to grow after 6 years of age. Therefore, in addition to the maturation of sensory [27] and attentional [1], [20], [28] mechanisms, which have been suggested to account for the continued development of auditory skill, we now propose that the prolonged development of the ability to utilize contextual cues that occur past the time frame of the single trial, also plays a role in the prolonged development of frequency discrimination.

### Frequency discrimination in preschool children

That frequency discrimination thresholds were poorer in children compared with adults is not surprising given previous reports of discrimination thresholds in school age [19], [20], [29] and preschool [18], [21] children. Whereas only approximately 20–25% of the children in the current study showed adult range frequency discrimination, the majority of our preschool participants (72/77) performed the frequency discrimination tasks reasonably, as determined by properties of the adaptive tracks such as number of reversals and false alarms rate (see Table 1). This is in contrast to the finding that only a minority of preschool children yielded measurable frequency discrimination thresholds [18].

Several differences in how frequency discrimination was assessed could potentially account for both why we were able to measure discrimination thresholds in the majority of children and why thresholds were so high and variable. First, in the current study children were required to decide whether the two tones on each trial were the same or not, a decision that is probably easier for them than deciding on the location of a different tone within a sequence of trails (an oddball procedure, [20]). Indeed, in a pilot phase to this study when an oddball procedure was administered to 15 kindergarten children, only 5 performed above chance level. Second, in the current study, on ‘different’ trials in the with-reference condition, the ‘different’ tone always occurred in a fixed temporal position (it was always the second tone), a factor known to positively affect the performance of school-age children [19] and adults [3]. This is in contrast to asking the children to select a tone pair in which the two tones were not identical as was done by Thompson and colleagues [18]. While measureable, discrimination thresholds of the preschoolers in the current study were much higher and more variable when compared to the performance of 6–7 y/o children measured with either an oddball procedure [28] or a 4 interval 2 alternatives forced-choice task in which children were asked to determine which tone pair contained two different tones [19], possibly reflecting less developed attention and memory skills in the younger children rather than poorer sensory resolution [20], [28]. It should also be noted that children in the current study were tested in a quite area within their preschool building, however, in our experience even those quite areas are noisier than a typical elementary school (or a lab) environment, thus also potentially contributing to the current pattern of high and highly variable thresholds.

### Anchoring in preschool children

The current data suggest that two types of processing that can contribute to performance on frequency discrimination tasks – direct trial by trial stimulus comparison and using or maintaining reference related information from previous trials, are immature among preschool children. Thus, the poorer performance of children than adults in the *no-reference* condition can be attributed to immature stimulus comparison processes. On the other hand, based on our definition of anchoring as the implicit ability to use information embedded in past stimuli to guide subsequent performance, the finding that children performed the *with-reference* condition significantly better than the *no-reference* one leads us to conclude that anchoring mechanisms are functional among the majority of preschool children, or their performance on the two conditions would have been equally poor. Because the threshold difference between the two conditions was generally smaller in children than in adults we also conclude that although present, the anchoring effect is still immature during the preschool period.

To the best of our knowledge, this is the first time that the use of context to facilitate auditory discrimination has been demonstrated in preschool children. The presence of an anchoring effect in the current data set is consistent with the observation made by Sutcliffe and Bishop that the lowest thresholds (approximately 0.14 octaves on average) in 6–7 y/o were achieved in a protocol in which 2 pairs of tones were presented on each trial (a total of 3 standard presentations and one presentation of the target) with the target occurring after the comparison [19]. Furthermore, in contrast to the observation that school-age children with non adult-like frequency discrimination did not benefit from the use of a consistent reference stimulus [29], here anchoring effects were characteristic of more than two thirds of the children across a wide range of discrimination thresholds (see Figure 1). This difference could arise due to the different assessment procedures used (same/different here, vs. oddball in the previous study [29]), or the environment in which the experiment was conducted (school vs. lab). A more likely explanation however is the fact that whereas the target tone in Halliday et al.'s study [29] could have been presented in either of 3 temporal intervals, the ‘different’ tones in the current study were presented in a fixed temporal position (the 2^nd^ tone) within the trial. This interpretation is consistent with the demonstration of Nahum et al. [3], that even in adults best performance, and thus presumably the strongest ‘anchoring’ effects is achieved when the target is presented at a fixed temporal position within a trial. It remains to be seen whether protocols that allow even more anchoring (e.g., by increasing the number of fixed references per trial) will increase the magnitude of the anchoring effect in young children.

It has been recently suggested that the use of ‘anchoring affording protocols’ triggers, in adult listeners, a ‘switch’ from comparison based to classification based performance. Thus, when the assessment protocol affords anchoring, instead of explicitly comparing the individual stimuli on each trial using working memory, listeners implicitly classify them as ‘reference’ or ‘non-reference’ based on a ‘reference template’ derived from stimuli presented on previous trials [3]. Here, we demonstrate that this is also true for children, albeit to a lesser extent. Whether this is so because children form less stable or faster to decay internal references or because they are unable to use the internal reference as efficiently as adults is beyond the scope of the present study and requires further investigation. One option that the current findings nonetheless help to refute is that immature anchoring derives from immature explicit working memory mechanisms because. We thus show that whereas working memory (assessed with an antonym production task) is significantly related to the ability to perform the *no-reference* task (in which the two tones on each trial must be explicitly compared to determine whether they are different), it was not significantly related to performance in the *with-reference* condition (see [5] for a similar pattern in adolescents). These data suggest that while similar working memory mechanisms may mediate performance in verbal and non-verbal auditory tasks, the ability to derive contextual information from ongoing stimulation is not directly related to the working memory components assessed here (phonological memory and central executive in Baddeley's model [30]).

Whereas the opportunity to anchor benefited the majority of children in the current study, this benefit was insufficient to yield adult like performance in the *with-reference* condition, suggesting that weaker anchoring is not the only cause of the immature performance of preschool children. It has previously been suggested that the immature performance of children on frequency discrimination results from their inability to sustain attention to the task throughout testing [20]. To the extent that lapses in attention should result not only in failing to detect the difference between two different tones but also in ‘false alarms’ (deciding that two identical tones are different), the current data do not provide evidence for more lapses of attention in children compared with adults (see Table 1).

### Perceptual anchoring and dyslexia

Perceptual anchoring of the type observed here among young children has been recognized for decades in adults (at least since the 1940's [8]), but some of the recent interest in the phenomenon stems from findings of abnormal anchoring in dyslexia [5]. Similarly to what we have previously observed in typically developing adolescents, the magnitude of the anchoring effect among typically developing preschool children was not correlated with early reading skills such as phonological awareness and letter knowledge. Determining whether impaired anchoring during preschool could play a causal role in the development of reading problems upon school entry requires further studies with children who are at risk of developing reading difficulties due to family history or the presence of language deficits. Nonetheless, that anchoring is present among the majority of preschool children suggests that the abnormal anchoring of individuals with dyslexia is more likely a result of truly deficient rather than of less developed anchoring mechanisms, because otherwise the deficit may have been expected to diminish by adolescence, which was not the case in our previous studies [5], [31]. Again, further developmental studies are required to resolve this issue.
